# Differential Gene Expression and Immune Cell Infiltration in Patients with Steroid-induced Necrosis of the Femoral Head

**DOI:** 10.2174/0118715303266951231206114153

**Published:** 2024-01-09

**Authors:** Guowu Ren, Jie Han, Jian Mo, Zhiwei Xu, Xinjian Feng, Feng Chen, Yukun Wu, Qinglin Peng

**Affiliations:** 1 Guangxi University of Traditional Chinese Medicine, Nanning City, Guangxi Zhuang Autonomous Region, 530001 China;; 2 Department of Orthopedics, Wenshan Prefecture Traditional Chinese Medicine Hospital, Yun Nan Region, 663100 China;; 3 Department of Orthopedics, Ruikang Hospital Affiliated to Guangxi University of Traditional Chinese Medicine, Nanning City, Guangxi Zhuang Autonomous Region, 530011 China

**Keywords:** Steroid-induced necrosis of the femoral head, bioinformatics, immune cells, cellular infiltration, NOD-like receptor, immunohistochemistry

## Abstract

**Objective:**

The study aimed to study the differential gene expression and immune cell infiltration in patients with steroid-induced necrosis of the femoral head (SANFH), identify the key genes and immune cells of SANFH, and explore the relationship between immune cells and SANFH.

**Methods:**

The high-throughput gene chip dataset GSE123568 was downloaded from the GEO database, and the differential gene expression was analyzed with the R language. The STRING database and Cytoscape software were used to analyze the protein interaction network and screen key genes, and enrichment analysis was carried out on key genes. The infiltration of immune cells in SANFH patients was analyzed and verified by immunohistochemistry.

**Results:**

EP300, TRAF6, STAT1, JAK1, CASP8, and JAK2 are key genes in the pathogenesis of SANFH, which mainly involve myeloid cell differentiation, cytokine-mediated signaling pathway, tumor necrosis factor-mediated signaling pathway, and cellular response to tumor necrosis factor through JAK-STAT, NOD-like receptor, toll-like receptor, and other signaling pathways, leading to the occurrence of diseases; immune infiltration and immunohistochemical results have shown the expression of memory B cells and activated dendritic cells as reduced in SANFH patients, while in the same SANFH samples, M1 macrophages have been positively correlated with monocytes, and neutrophils have been negatively correlated with monocytes expression.

**Conclusion:**

EP300, TRAF6, STAT1, JAK1, CASP8, and JAK2 have exhibited significant differences in SANFH (spontaneous osteonecrosis of the femoral head). Memory B cells, activated dendritic cells, M1 macrophages, monocytes, and neutrophils have shown abnormal expression in SANFH.

## 1 INTRODUCTION

Avascular necrosis of the femoral head (ANFH or ONFH) is a debilitating disease that primarily affects young and middle-aged individuals. According to statistics, the total number of diagnosed patients worldwide has exceeded 30 million, with over 8 million in China alone. Additionally, approximately 75,000 to 150,000 new cases are reported each year [1, 2]. Among the various types of ONFH, steroid-associated necrosis of the femoral head (SANFH) is the most common. It is estimated that there are around 80,000 individuals aged 12 or above in China affected by femoral head necrosis, with steroid-induced cases accounting for 51% and showing an increasing trend over the years. SANFH primarily affects young to middle-aged individuals, with a higher incidence rate among males compared to females [1-3]. However, the complex pathogenesis of SANFH has not been fully elucidated. The main recognized theories regarding its development include the vascular thromboembolic theory, lipid metabolism disorder theory, osteoporosis theory, and cellular apoptosis theory [4-6]. With the rise of bone immunology, increasing research suggests shared molecules and interactions between the immune system and the skeletal system. Studies have indicated that bone marrow tissue is rich in hematopoietic stem cells and mature immune cells, and that the activation or inhibition of immune cell surface factors (RANKL) and secreted immune factors (IL-1, IL-6, M-CSF, TGF-β, TNF, CD80, *etc*.) in SANFH interact with bone tissue cells, such as osteoblasts, osteoclasts, and bone microvascular endothelial cells, ultimately leading to an imbalance in bone homeostasis [7]. Currently, there is limited research on the molecular mechanisms of immune cells in SANFH. Immune cells play a crucial role in the occurrence and development of SANFH. Targeting immune cells in the SANFH region and key factors and proteins in their regulatory pathways may contribute to immune-targeted and personalized therapies, helping to regulate bone balance and prevent or reduce further damage to the femoral head caused by immune dysregulation. This approach may become a viable method for delaying the occurrence and progression of SANFH, and even reversing the pathology. Therefore, from an immunological perspective, evaluating the differences in immune infiltrating cell composition in SANFH is of significant value in elucidating its molecular mechanisms and identifying molecular markers associated with immune infiltration.

In this study, gene expression profile data of serum samples from SANFH patients were downloaded from the Gene Expression Omnibus (GEO) dataset. Differential gene expression analysis and functional signaling pathway analysis were conducted to identify the genes and signaling pathways associated with SANFH. Additionally, the expression levels of 22 immune cell types in SANFH were analyzed using the CIBERSORT software package in R. The interaction between differential genes, signaling pathways, and immune cells in SANFH was further investigated. Finally, a SANFH mouse model was established to validate immune cell infiltration in SANFH through immunohistochemistry. These findings provide a new approach for studying the molecular immune mechanisms of SANFH.

## 2 MATERIALS AND METHODS

### 2.1 SANFH Chip Screening

“Steroid-induced osteonecrosis of the femoral head”, “human”, and “peripheral blood” were used as keywords to search the related chips of SANFH in the GEO database, and the matrix file numbered GSE123568 and the gene annotation file of GPL15207 were obtained. The chip contained 40 peripheral serum samples, including 30 SANFH patients and 10 healthy controls.

### 2.2 SANFH Chip Data Analysis

Gene re-annotation was performed on the data using Perl language (version 5.30.0.1) to obtain gene expression profiling datasets of SANFH patients and healthy controls, and the genes were corrected and differentially analyzed using the limma package of the R language (version 4.1.2). Using *p* <0.05 and |log2 fold change (FC)|>0.5 as filter conditions, the differentially expressed genes were screened out, and the pheatmap package in R language was used to draw a heat map for the differential genes.

### 2.3 Construction of Protein Interaction Network

To further explore the protein interaction relationship of SANFH, the differential genes were introduced into the protein-protein interaction (PPI) analysis database STRING (https://string-db.org/); the study species was limited to “Homo sapiens”, and the connection score was set to > 0.99 to obtain the protein interaction relationship. The obtained results were imported into Cytoscape software (version 3.7.2), the tool “NetworkAnalyzer” was used to visualize and construct a protein interaction network, and the tool “CytoHubba” was used to screen out the key genes according to the degree value.

### 2.4 Functional Enrichment Analysis of Key Genes

With the clusterProfiler package in the R language, Gene Ontology (GO) and Kyoto Encyclopedia of Genes and Genomes (KEGG) pathway enrichment analyses were performed on key genes to study the main biological functions and signaling pathways involved in the occurrence and development of SANFH. Finally, the ggplot2 package of the R language was used to draw GO and KEGG enrichment analysis bubble charts.

### 2.5 Immunocytometry

With the deconvolution calculation of the R language CIBERSORT (https://cibersort.stanford.edu/) package and the 22 immune cell transcription feature matrices provided, 1000 simulations of expression profiles were performed to calculate the proportion of different immune cells in the peripheral blood of SANFH patients and analyze the differences of immune cells in peripheral blood of SANFH patients and healthy controls to obtain the infiltration and distribution of immune cells. R language packages, such as barplot, pheatmap, corrplot, vioplo, and ggplot2 were then used to draw a ratio histogram for each immune cell, immune cell expression heatmap, violin plot, and correlation heatmap.

### 2.6 Animal Experiment

#### 2.6.1 Experimental Animals

A total of 12 healthy female C57BL/6 wild-type mice, weighing 200 ± 20 g, were used as experimental animals. The animals were housed in the animal facility of the Scientific Experimental Center at Guangxi University of Traditional Chinese Medicine under standard conditions of temperature (22 ± 3°C) and humidity (60 ± 10%). All experimental animals were subjected to a one-week acclimation period before the start of the experiment. The experimental protocol was approved by the Ethics Committee for Animal Experiments at Guangxi University of Traditional Chinese Medicine. The study was conducted in accordance with the guidelines outlined in the “Animal Research: Reporting of *In vivo* Experiments” (ARRIVE) guidelines, and adhered to the principles laid out in the 1986 Animals (Scientific Procedures) Act of the United Kingdom, relevant guidelines, the European Union Directive 2010/63/EU on the protection of animals used for scientific purposes, and the Guide for the Care and Use of Laboratory Animals published by the National Institutes of Health (NIH publication No. 8023, revised in 1987).

#### 2.6.2 Experimental Design

Prior to the experiment, all groups of rats were subjected to a 12-hour fasting period. The mice were randomly divided into two groups, with 6 mice in each group. The experimental group was induced with steroid-induced avascular necrosis of the femoral head (SANFH) by subcutaneous injection of 21 mg/kg/d methylprednisolone (MPS, Pfizer, Rijksweg, NL). The control group received an equivalent volume of saline solution. The administration of the drug or saline lasted for 4 weeks. After the final administration, the rats in all groups underwent a 12-hour fasting period and were euthanized the next morning at 9 am. The femoral heads were dissected and placed on a gridded paper for observation and photographic documentation of the gross morphology of the femoral head. The diagnosis of bone necrosis was based on the presence of lacunae or dead bone cells in the trabeculae of the bone, accompanied by necrosis of the surrounding bone marrow.

#### 2.6.3 Histopathology

Femur specimens were fixed in 10% neutral formalin for 24 hours at room temperature, then decalcified in 10% EDTA-Tris solution (Servicebio, Wu Han, China) for 4 weeks at room temperature (decalcification solution was changed every 3 days), and embedded in paraffin. The sample was cut into 5 µm sections, dewaxed in xylene and environmentally friendly dewaxing transparent solution, rehydrated in fractionated series ethanol, and rinsed in distilled water. Hematoxylin and eosin (HE) staining was performed on tissue sections to assess tissue structure and damage status.

#### 2.6.4 Immunohistochemical Analysis

Decalcified samples in each group were dewaxed with xylene and hydrated with graded alcohol. The samples were stored at room temperature for 10 min, and the activity of endogenous peroxidase was blocked by 3% H_2_O_2_. Sections were then incubated overnight with anti-CD31 antibody (diluted concentration 1:300), CD34 antibody (diluted concentration 1:300), and vWF antibody (diluted concentration 1:300), respectively, followed by incubation with secondary antibody for 30 min. Sections were then incubated for 5 min with DAB peroxidase substrate. Counterstaining was performed with hematoxylin, which was followed by dehydration. All sections were washed with xylene, coverslips were sealed and stained with a fluorescence microscope (E100; Nikon, Hamburg, Germany), and immunohistochemical stained images were analyzed using the software ImagePro Plus, as shown in Fig. (**1**).

### 2.7 Statistical Analysis

Quantitative data have been presented as mean ± standard deviation (SD). A paired t-test was used to analyze the phenotypic differences between groups and estimate the relationship between each genotype and the risk of SANFH. The odds ratio (OR), 95% confidence interval (CI), and p-value for SANFH were calculated. Data analysis was performed using SPSS 23.0 software (SPSS, USA). A significance level of *p* <0.05 was considered significant.

## 3 RESULTS

### 3.1 Analysis of SANFH Differential Genes

The chips were re-annotated with tools, such as the Perl and R language, and then differences were analyzed. The results showed that compared to healthy controls, SANFH patients had a total of 1836 significantly altered genes, of which 1088 were up-regulated and 748 were down-regulated. The 20 genes with the most significant differences among the up-regulated and down-regulated genes were selected, respectively, to draw a differential gene heat map, as shown in Fig. (**2**).

### 3.2 PPI Network

PPI network was constructed with the help of the STRING database and Cytoscape software, as shown in Fig. (**3**). A total of 148 nodes and 212 edges have been involved in the graph, and the top 6 protein genes, EP300, TRAF6, STAT1, JAK1, CASP8, and JAK2, have been screened out according to the degree value. These protein genes with larger degrees play a key role in the entire network and may be key genes in the occurrence and development of SANFH. The basic information is shown in Table **1**.

### 3.3 GO Enrichment Analysis and KEGG Enrichment Analysis

A total of 589 items were identified in the GO enrichment analysis of the functional process of key genes, of which 510 represented biological processes, mainly involving myeloid cell differentiation, cytokine-mediated signaling pathway, tumor necrosis factor-mediated signaling pathway, cellular response to tumor necrosis factor, *etc*.; 13 items have represented cellular components, mainly involving membrane raft, membrane microdomain, focal adhesion, and cell-substrate junction; 66 have represented molecular functions, mainly involving cytokine receptor binding, ubiquitin-like protein ligase binding, tumor necrosis factor receptor binding, and tumor necrosis factor receptor superfamily binding. The results of GO analysis have shown biological processes, cellular components, and molecular functions as closely related to the occurrence and development of SANFH, as shown in Fig. (**4**). A total of 43 key targets were identified by KEGG enrichment analysis, mainly involving the JAK-STAT signaling pathway, NOD-like receptor signaling pathway, and toll-like receptor signaling pathway, as shown in Fig. (**5**).

### 3.4 Distribution of Immune Cell Infiltration

22 types of immune cells have been found to be involved in this study, including naive B cells, memory B cells, plasma cells, CD8 T cells, CD4 naive T cells, CD4 memory-resting T cells, CD4 memory-activated T cells, follicular helper T cells, regulatory T cells (Tregs), γδ T cells, resting NK cells, activated NK cells, monocytes, M0 macrophages, M1 macrophages, M2 macrophages, resting dendritic cells, activated dendritic cells, resting mast cells, activated mast cells, eosinophils, and neutrophils. In the R language, the barplot package was used to draw a histogram, and the specific proportions of different immune cells in each sample could be observed, as shown in Fig. (**6**). Then, the pheatmap package was used to draw a heat map for the matrix data of immune cells and samples; memory B cells, CD8 T cells, CD4 memory-activated T cells, gamma delta T cells, resting NK cells, activated NK cells, monocytes, activated dendritic cells, and activated mast cells were relatively highly expressed in healthy controls. The expression of naive B cells, CD4 naive T cells, regulatory T cells (Tregs), M0 macrophages, resting mast cells, and neutrophils was relatively high in SANFH patients, as shown in Fig. (**7**).

### 3.5 Correlation Analysis between SANFH Immune Cells

Through the analysis of the correlation between immune cells in SANFH samples in this study, it was found that the correlation coefficient between macrophages M1 and monocytes was the highest, *i.e*., 0.53, which means that M1 macrophages were positively correlated with monocytes in the same SANFH sample. In the same sample, neutrophils and monocytes had the lowest expression value of -0.56, indicating the expression of neutrophils and monocytes as negatively correlated in the same SANFH sample, as shown in Fig. (**8**).

### 3.6 Difference Analysis of Immune Infiltration between SANFH Patients and Healthy Controls

Vioplot package of R language was used to draw violin diagrams. By analyzing the differences in immune infiltrating cells between SANFH patients and healthy controls, it was found that the expressions of B cells memory and dendritic cells activated were decreased in SANFH patients (*p* <0.05;Fig. (**9**)).

### 3.7 Histopathology of SANFH

In the control group, the trabecular bone structure was complete, the shape was regular and neat, and the continuity was good; the osteocytes were evenly stained, the structure was clear, and the shape was normal. Osteoblasts were seen, and only a few empty bone lacunae, fat vacuoles, and osteoclasts were seen. The trabecular bone in the model group was irregular in shape, sparse, disordered, thinned, and even interrupted in continuity; there were destroyed cell structure, cell atrophy, cell agglomeration, and pyknosis. Empty bone lacuna, fat vacuoles, and osteoclasts were common, as shown in Fig. (**10**).

### 3.8 SANFH Immunohistochemistry

**Fig. (11):**
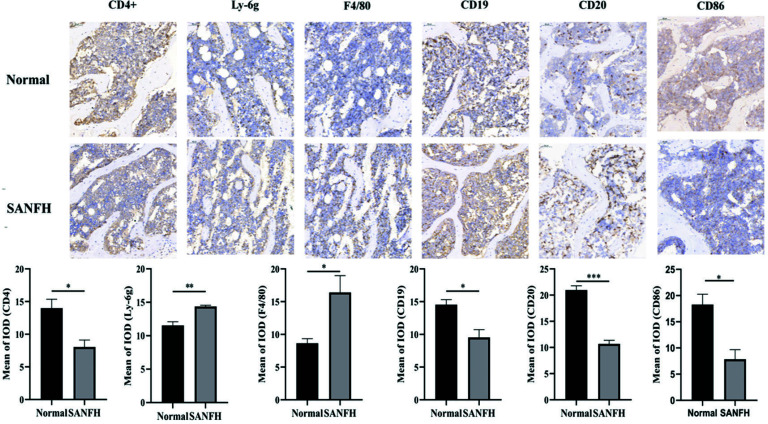
Immunohistochemical images and statistical analysis of mouse femoral head. Abnormal expression of SANFH in immune cells (*indicates *p* <0.05; **indicates *p* <0.01).

To verify the mechanism of immune cell infiltration in SANFH, immunohistochemistry was performed to examine the expression of Th cells (CD4), B cells (CD19), M1 macrophages (F4/80), neutrophils (Ly-6g), memory B cells (CD20), and dendritic cells (CD86) in femoral head tissue. Compared to the normal group, the expression of Th cells, B cells, memory B cells, and dendritic cells in the SANFH group was decreased, while the expression of M1 macrophages and neutrophils was increased, with *p* < 0.05 (Fig. **11**).

## 4 DISCUSSION

The inability to diagnose steroid-induced necrosis of the femoral head early results in a delay in hip joint preservation treatment. While magnetic resonance imaging is commonly used as an early diagnostic method for this disease, it is not suitable for screening all patients undergoing GC treatment due to the high cost and time required for scanning [8]. Therefore, the identification of key biomarkers for SANFH is crucial for its early diagnosis. In recent years, researchers have explored some key biological markers for SANFH, such as I-type collagen cross-linked C-terminal peptide and amino-terminal peptide of type I collagen [9]. However, it has been found that both of these markers are also associated with the development of osteoporosis, limiting their diagnostic value for SANFH [10]. Therefore, it is necessary to identify promising biomarkers for SANFH to improve its diagnosis and treatment. In our study, we have attempted to identify potential key genes related to SANFH. GO and KEGG enrichment analyses were performed for bioinformatics evaluation, and we have identified key hub genes in our study. The data have been extracted from the GSE123568 chip in this study. Using bioinformatics analysis, we have identified 1088 upregulated genes and 748 downregulated genes between SANFH patients and healthy controls. To explore the key genes, signaling pathways, and immune cell mechanisms involved in SANFH, we have screened the key genes in the protein-protein interaction network and conducted enrichment analysis. The results have shown the protein genes EP300, TRAF6, STAT1, JAK1, CASP8, and JAK2 to be upregulated in the serum of SANFH patients, and they have been mainly enriched in the JAK-STAT, NOD-like receptor, and toll-like receptor signaling pathways.

### 4.1 JAK1, JAK2, STAT1, and JAK-STAT Signaling

JAKs belong to a family of non-receptor protein tyrosine kinases, including JAK1, JAK2, JAK3, and TYK2. STATs are potential cytoplasmic transcription factors, including 7 subtypes STAT1-6. After the immune factor binds with related receptors, the receptor subunit domain in the cytoplasm binds to JAK, and the activated JAK phosphorylates STAT substrates [11]. After that, STATs interact with SH2 domains to form dimerized STATs, and the dimerized STATs are transferred into the nucleus to activate or inhibit the transcription of target genes to complete the regulation of cells. Studies have shown that the expression of IL-9 (major secretory cells: mast cells, helper T cells) and IL-21 (major secretory cells: helper T cells) in the serum or necrotic bone tissue of patients with SANFH is increased, and IL-9 and IL- 21 activate JAK1 and JAK3 after binding to their receptors, resulting in the phosphorylation of STAT1 and STAT3. STAT1 and STAT3 are then transferred to the nucleus to activate targeted genes, and finally promote chondrocytes to secrete COX-2, IL-1β, and matrix metalloproteinase-13 (MMP-13), and other substances, leading to hip inflammation, pain, and bone structure destruction [12, 13], as shown in Fig. (**12**). In addition, JAK-STAT signaling also corresponds to the activation of IFN-β (major secretory cells: lymphocytes (NK cells, B cells, and T cells) and macrophages). Kim *et al*. suggest that IFN-β inhibits the activation of osteoclasts by activating the JAK-STAT pathway, thereby improving trabecular bone destruction in osteonecrosis [14, 15].

### 4.2 Toll-like Receptor

Toll-like receptors (TLRs) belong to the family of pattern recognition receptors (PRRs) and are primarily expressed in monocytes/macrophages and dendritic cells. TLRs need to form heterodimers or homodimers for activation, such as TLR1 or TLR6 form heterodimers with TLR2, while TLR4 typically forms homodimers [16-18]. It has been shown that steroids can induce TLR4 activation. Once activated, TLRs recruit MyD88 (myeloid differentiation primary response 88) to interleukin-1 receptor-associated kinase 1 (IRAK1). Phosphorylated IRAK1 then recruits TRAF6 to the toll receptor complex, which is followed by the dissociation of phosphorylated IRAK1 and TRAF6 from the receptor and their binding to proteins, like TAK1, TAB1, TAB2, *etc*. TAK1 induces downstream IκB kinase/NF-κB signaling, ultimately leading to osteoclast activation, lipid cell generation, and elevated levels of inflammatory factors, such as IL-6, IL-10, and TNF-α [18-22], as shown in Fig. (**13**).

### 4.3 NOD-like Receptor

Nucleotide-binding oligomerization domain-like receptors (NLRs), also known as NOD-like receptors, are another type of PRR. Although there is currently no direct research on the NLR signaling pathway in steroid-induced avascular necrosis of the femoral head (SANFH), numerous studies have indicated the involvement of NLRs in inflammation and bone resorption. Both pathogen-associated molecular patterns (PAMPs) and damage-associated molecular patterns (DAMPs) can activate NLR signaling [23, 24]. Previous studies have shown elevated expression of TNF-α, a PAMP, in the serum of SANFH patients. The implantation of prosthetic particles in the femoral head and the degradation of the articular cartilage matrix are examples of DAMPs. Prosthetic particles can induce the activation of NOD-like receptor pyrin domain-containing 3 (NLRP3) in macrophages, leading to the release of IL-1β and HMGB1, promoting local inflammation and bone resorption around the femoral head [25, 26]. Additionally, NLRP3 is highly expressed during osteoclast differentiation and is associated with NF-κB signaling [27-29], as shown in Fig. (**13**).

EP300 functions as a transcriptional coactivator and histone acetyltransferase that mediates the life processes in bone cells and endothelial cells. In osteoblasts, EP300 promotes the activity and stability of the osteogenic factor Runx2 through its acetyltransferase activity [30]. In osteoclasts, EP300 acts cooperatively with osteoclastic differentiation factors, c-Fos and NFATc1, to exert co-activating effects [31]. Under hypoxic conditions, endothelial cells compensate by expressing HIF-α, which, together with EP300, activates downstream target VEGF to promote endothelial cell proliferation and migration [32].

SANFH exhibits various pathological changes similar to endothelial cell injury, osteoclast activation, and decreased bone formation under ischemia and hypoxia conditions. Based on these pathological changes, it is speculated that EP300 may play a key role in mediating SANFH. CASP8, a member of the cysteine aspartate protease family, plays a central role in the execution phase of cell apoptosis. Studies on SANFH suggest that under the dual action of hormones and hypoxia, CASP8 mediates changes in the mitochondrial permeability of osteoblasts, leading to osteoblast apoptosis [33].

The analysis of immune cell distribution revealed that naive B cells, CD4 naive T cells, regulatory T cells (Tregs), M0 macrophages, resting mast cells, and neutrophils are relatively highly expressed in patients with SANFH. Correlation analysis showed a positive correlation between M1 macrophages and monocytes expression and a negative correlation between neutrophils and monocytes expression. Bone marrow tissue is rich in myeloid cells (which can differentiate into macrophages and granulocytes), lymphoid cells (including T cells, B cells, and NK cells), and pluripotent stem cells. Studies have shown that B cells and CD4+ T cells (helper T cells, Th) highly express RANKL during differentiation. They promote osteoclast differentiation by interacting with RANK on the surface of osteoclast precursor cells through cell-cell contact [34, 35]. Additionally, GM-CSF, TNF, and IL-1 secreted by B cells, Th cells, and macrophages act on macrophages, promoting inflammation infiltration and osteoclast formation [36]. Vicaş *et al.* found a significant infiltration of B cells (mainly distributed in the necrotic conjunctive tissue), T cells (mainly distributed around blood vessels), and macrophages (mainly distributed at the edge of ischemic necrotic areas) in the necrotic area of ONFH [37]. Therefore, immune cells play a role in mediating the occurrence and development of ONFH.

### 4.4 CD4+T Cells and ONFH

CD4+ T cells, also known as helper T cells (Th), can differentiate into different subgroups, such as Th1, Th2, Th9, Th17, Th22, Treg, and follicular helper T (TFH) cells in response to different stimuli. These subsets of cells secrete their own cytokines and perform specific functions to complete the entire immune response [23]. The role of Th cells in ONFH has been mainly studied in clinical research. However, the number of T cells in the blood of ONFH patients is decreased (including CD3+ T cells, Ts cells, Th cells, and CD8 T cells), and the expression of TGF-β (an executive factor of Treg cells) in the necrotic area is significantly reduced [38, 39]. Bone marrow mesenchymal stem cells (BMSCs) and osteoblasts express various subtypes of TGF-β receptors (TGFRs). The binding of TGF-β to TGFRs induces the differentiation of BMSCs into osteoblasts, promoting bone formation [40]. A large number of CD4+ T cells, including the Th17 subset, are expressed in the synovium of ONFH patients, and IL-17 is positively correlated with patient symptoms [41, 42]. During the differentiation process of Th17 cells, they highly express RANKL on their surface, inducing the generation of RANK signals on osteoclasts and promoting osteoclast formation. IL-17 directly enhances the bone resorption capacity of osteoclasts and indirectly promotes osteoclast formation by inducing the expression of RANKL on osteoblasts [43, 44], as shown in Fig. (**14**). Firstly, the study of T cells in ONFH mainly focuses on non-traumatic ONFH, which leads to a lack of specific classification and cannot fully represent steroid-induced or alcohol-induced ONFH. Secondly, the specimens used in the research mainly include synovium and blood from patients, which cannot represent the pathological changes in bone tissue. However, our SANFH immunohistochemistry results showed a decrease in the total number of Th cells, confirming the presence of functional abnormalities of Th cells in SANFH.

### 4.5 Neutrophils and Monocytes

Studies by Nonokawa *et al.* have shown that glucocorticoid (GC)-induced platelet activation promotes the formation of neutrophil extracellular traps (NETs), which are distributed around the small blood vessels in the area of femoral head necrosis, further contributing to ischemia [45]. In patients with GC-induced ANFH, there is an increased number of macrophages in the pathological bone tissue compared to trauma-induced ANFH [46]. Imbalanced polarization of macrophages is also involved in the pathogenesis of SANFH, as studies have shown that as the disease progresses (from Ficat stage III to IV), the numbers of M1 and M2 macrophages decrease while the M1/M2 ratio gradually increases, accompanied by increased secretion of IL-6 and IL-1β (cytokines produced by M1 macrophages) [47]. The results of this study are similar, indicating a significant increase in M1 macrophages in the necrotic bone tissue. Macrophages can stimulate neutrophil differentiation by secreting granulocyte colony-stimulating factor (G-CSF), and neutrophils interact with macrophages to induce the production of pro-inflammatory cytokines/chemokines (such as TNFα and CXCL1/CXCL2), promoting local inflammation. Neutrophils can also release NETs induced by damage-associated molecular patterns (DAMPs), further promoting inflammation [48-50]. Under physiological conditions, neutrophils undergo apoptosis after releasing NETs, and macrophages are responsible for phagocytosing apoptotic neutrophils and degrading NETs [51]. However, under the pathological conditions of SANFH, the interaction between neutrophils and macrophages may promote local inflammation. Phipps *et al.* found that neutrophils and macrophages accumulated synchronously in a surgical model of femoral head necrosis in mice, which has led to elevated levels of TNFα [52]. Furthermore, as SANFH progresses, the number of macrophages decreases, weakening their ability to clear apoptotic neutrophils and degrade NETs [53-56], as shown in Fig. (**15**).

Differential analysis of immune infiltration has revealed that memory B cells and activated dendritic cells are reduced in SANFH patients. B cells differentiate and develop from hematopoietic stem cells in the bone marrow, and they produce antibody-mediated humoral immune responses. CD19+ B cells represent the total B cell population, while the formation of B cells covers naive (IgD +) and activated B cells (CD95+), plasma cells (CD38+), and memory B cells (CD20+CD27+/-), which can be re-stimulated to differentiate into plasma cells [57-59]. In the blood of SANFH patients, naive B cells, memory B cells, and total B cells were reduced, while activated B cells and plasma cells were increased, and memory B cells were negatively correlated with the number of plasma cells; therefore, the formation of B cells was reduced in SANFH, but there may be persistent stimulation that reactivates memory B cells to differentiate into plasma cells to mediate inflammation [60]. Differentiation of B cell precursors requires contact with osteoblast-expressed CXCL12 (SDF-1), and activation of the PTH/PTH-related peptide receptor (PPR) promotes osteoblast expression of CXCL12. However, the expression of CXCL12 in the blood of SANFH was decreased, and the expression of PTH in osteoblasts under GC treatment was also decreased, while the PPR was increased and showed a trend of apoptosis [61-64]. The functional impairment of osteoblasts may lead to a decrease in the number of B cells. In addition, toll-like receptors are also expressed on B-cell receptors, and PAMPs can induce B-cell proliferation and activation by activating toll-like receptors [65, 66]. The nuclear debris generated during the execution phase of apoptosis moves to the cell surface to form surface protrusions (blebs), while self-antigens are covalently modified and blebs acting as antigen-mediated B cell activation are redistributed [67, 68]. ONFH has the potential to activate PAMPs/toll-like receptors and many apoptotic cells (including neutrophils, osteoblasts, and endothelial cells) [69], as shown in Fig. (**16**).

### 4.6 Dendritic Cells

Dendritic cells and osteoclasts have a common progenitor cell and share the same RANK and toll receptors, playing crucial roles in immune response and bone resorption, respectively. Under the stimulation of RANKL and M-CSF or IL-17, dendritic cells can differentiate into osteoclasts. Osteoclasts derived from dendritic cells can present bone matrix and bone fragments generated from bone resorption to CD4+ T cells, further amplifying the inflammatory cascade reaction [70, 71]. In addition, the cytokines secreted by dendritic cells have dual effects on osteoclasts. IFN-α, IFN-β, and IFN-γ can interfere with the RANKL/RANK signaling of osteoclasts, while TNF-α and IL-17 promote the formation of osteoclasts [72, 73]. Tateda *et al.* found that the number of dendritic cells in GC-induced ONFH rats was highest at 12 hours, gradually decreasing thereafter and being lower than the normal group, but IFN-α showed continuous accumulation [74, 75]. Immunohistochemistry in this study also supported this result, showing a decrease in the number of dendritic cells in the necrotic tissue of SANFH, possibly indicating an increased differentiation of dendritic cells into osteoclasts in SANFH. Moreover, the pro-inflammatory capacity increased while the anti-inflammatory function decreased, as shown in Fig. (**17**).

## CONCLUSION

In conclusion, EP300, TRAF6, STAT1, JAK1, CASP8, and JAK2 exhibit significant differences in SANFH (spontaneous osteonecrosis of the femoral head). Memory B cells, activated dendritic cells, M1 macrophages, monocytes, and neutrophils show abnormal expression in SANFH. Limitations of the current study include sample size limitations, complexity of data interpretation, and limitations of study design; thus, future prospects can include enlarging sample size, multi-component joint analysis, establishing animal model and cell experiment verification, individualized diagnosis and treatment strategy, *etc*.

Exploring the abnormal mechanism of “bone immunity” in SANFH is of great significance in developing therapeutic measures for SANFH, such as reducing foreign body reactions after prosthesis implantation from an immunological perspective, the mechanism of survival and action of cell transplantation, and targeting inhibitors or the effect of activators on the overall intra-osseous microenvironment. Therefore, improving the imbalance of bone cells and immune cells in SANFH may be one of the directions for future research.

## Figures and Tables

**Fig. (1):**
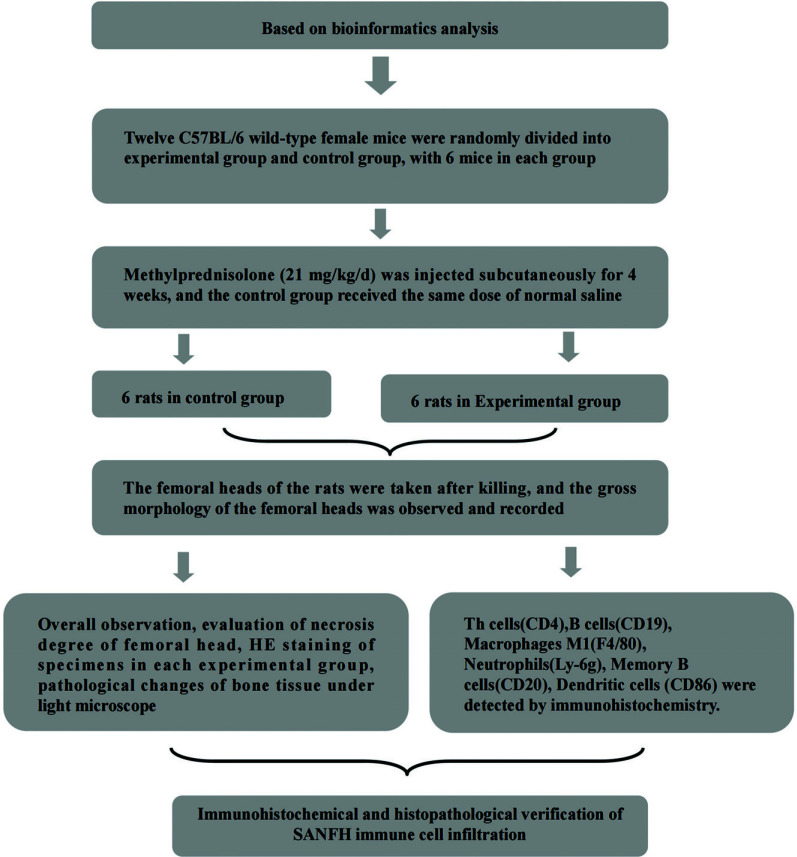
Flow chart of animal experiment.

**Fig. (2):**
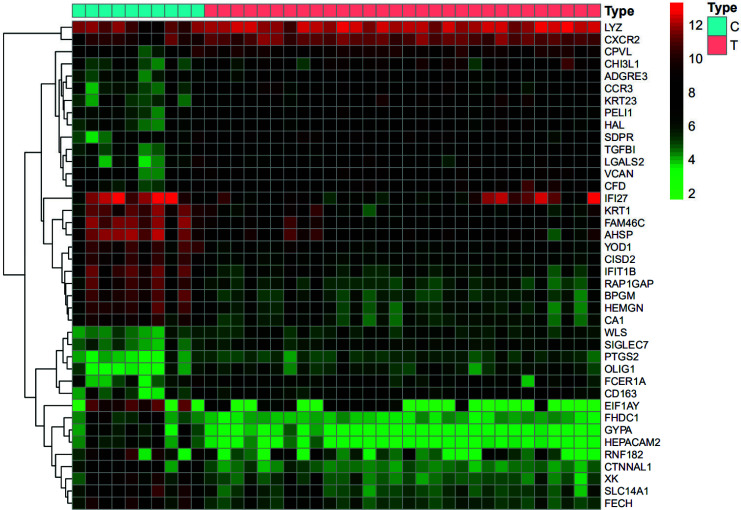
Differential gene heat map. The left vertical axis presents the cluster analysis of the differential genes, and the right vertical axis shows the differential genes. Red represents high relative expression, brighter red represents more significant high relative expression, green represents low relative expression, brighter green represents more significant low relative expression, and black represents no significant difference in relative expression. The upper blue part represents peripheral blood samples from healthy controls and the red part represents peripheral blood samples from SANFH patients.

**Fig. (3):**
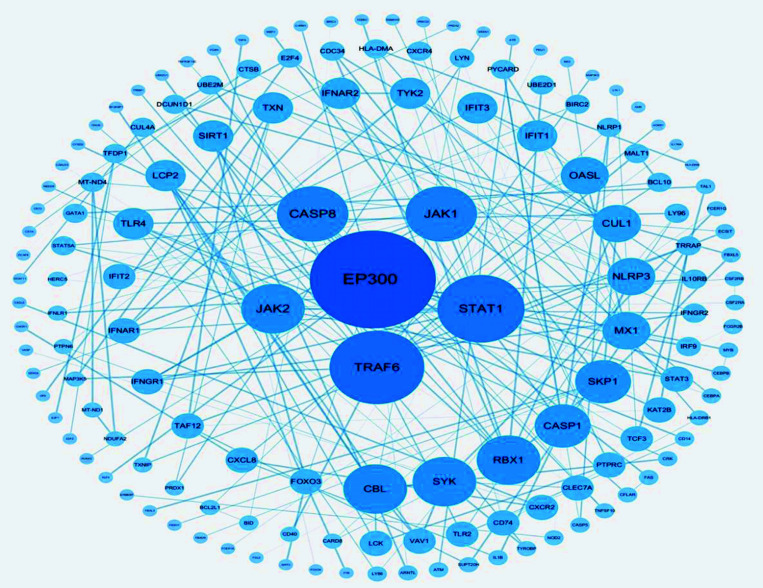
Protein interaction network. The node represents the protein gene, and the line connecting the nodes represents the interaction between each node; the larger the node and the darker the color, the greater the degree value. The thickness of the edge reflects the connection score, and the thicker the edge, the closer the interaction relationship between proteins.

**Fig. (4):**
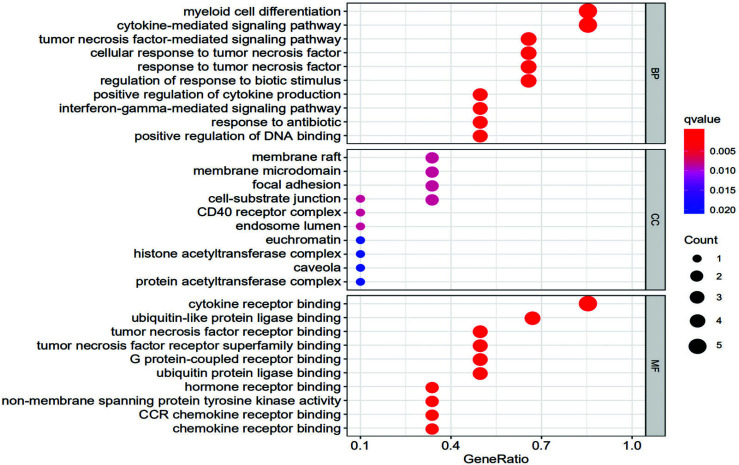
GO enrichment analysis of key genes bubble diagram. The vertical axis represents the GO enrichment analysis, and the horizontal axis represents the proportion of enriched genes to the total human genes. The redder the color of the bubbles, the more significant the enrichment degree is, and the larger the bubbles, the more genes are enriched in this item.

**Fig. (5):**
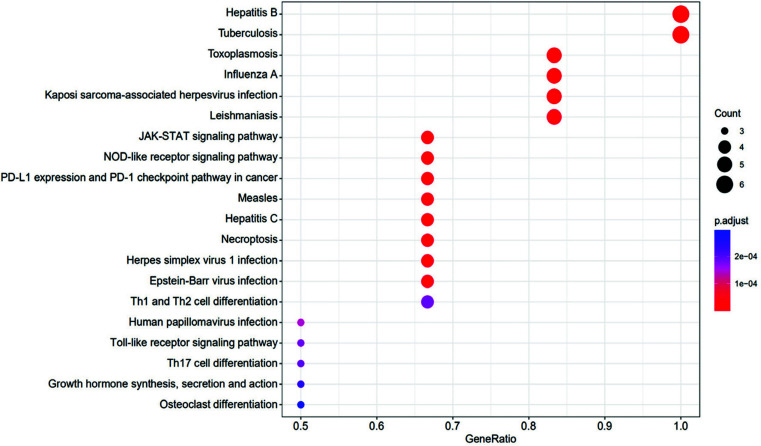
KEGG enrichment analysis of key genes bubble diagram. The vertical axis represents the pathway, and the horizontal axis represents the proportion of enriched genes in the total human genes. The redder the color of the bubbles, the more significant the enrichment degree is, and the larger the bubbles, the more genes are enriched in this item.

**Fig. (6):**
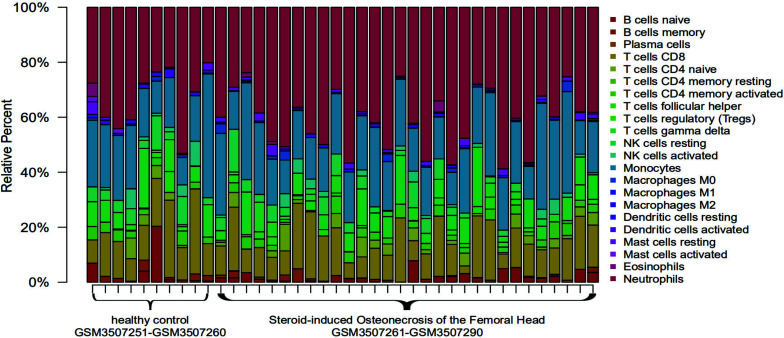
Histogram of distribution of immune cells in different samples.

**Fig. (7):**
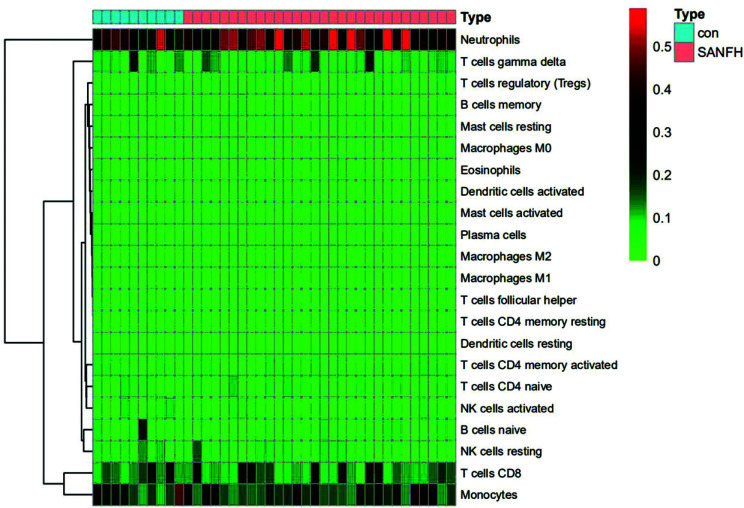
Expression levels of immune cells in different samples.

**Fig. (8):**
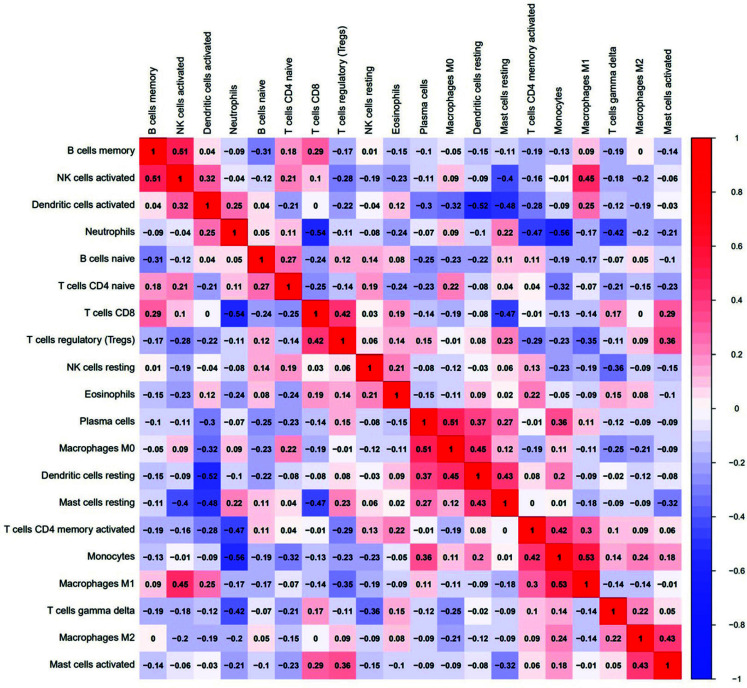
Heatmap of correlation between immune cells.

**Fig. (9):**
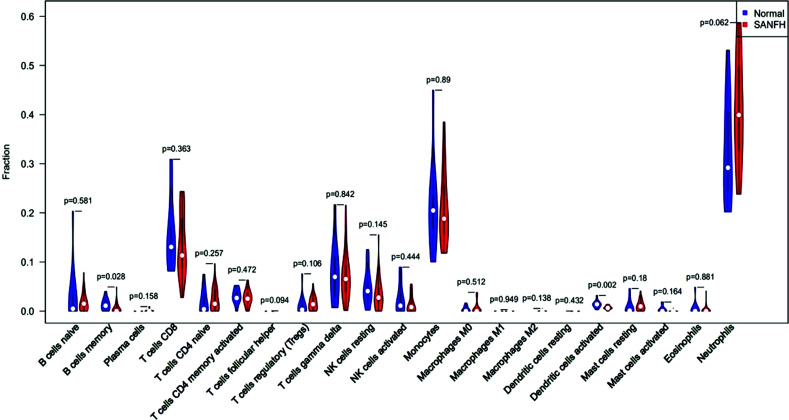
Comparison of immune cells between SANFH patients and healthy controls.

**Fig. (10):**
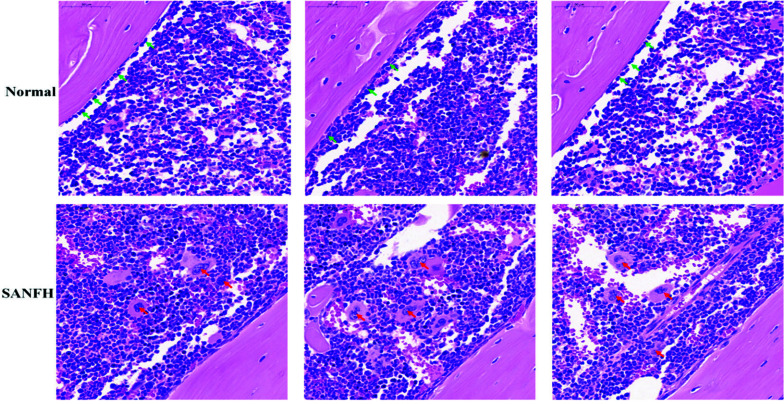
HE staining image of mouse femoral head. 50× magnification; green arrows represent osteoblasts and red arrows indicate osteoclasts.

**Fig. (12):**
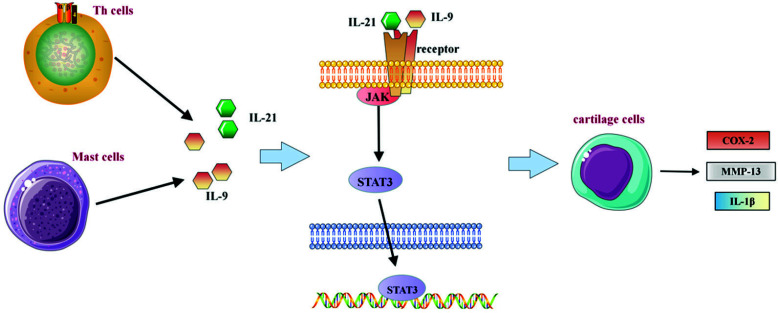
Diagrammatic representation of the mechanism of action of JAK-STAT signaling in SANFH.

**Fig. (13):**
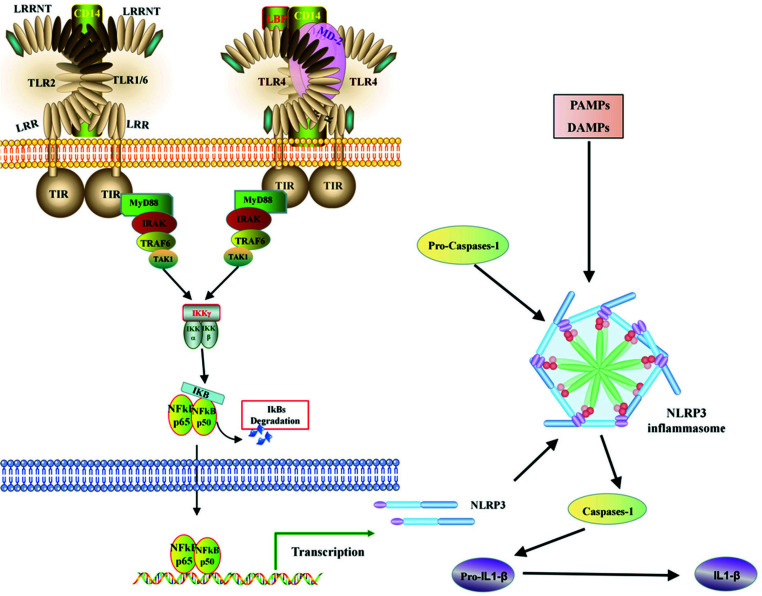
Diagrammatic representation of the mechanism of NOD and toll signaling in SANFH.

**Fig. (14):**
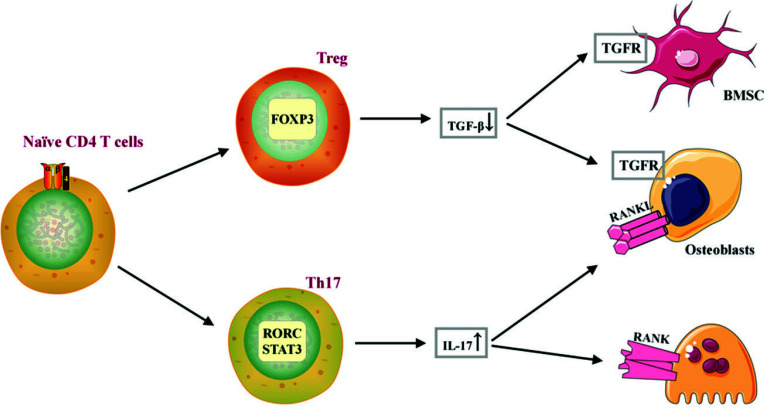
The mechanism of action of Th cells in SANFH.

**Fig. (15):**
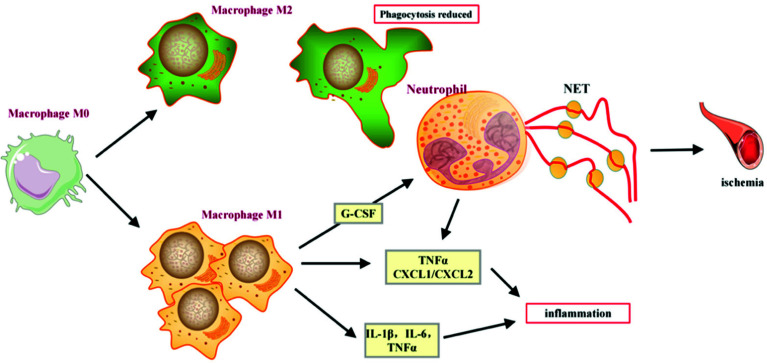
The mechanism of action of neutrophils and monocytes in SANFH.

**Fig. (16):**
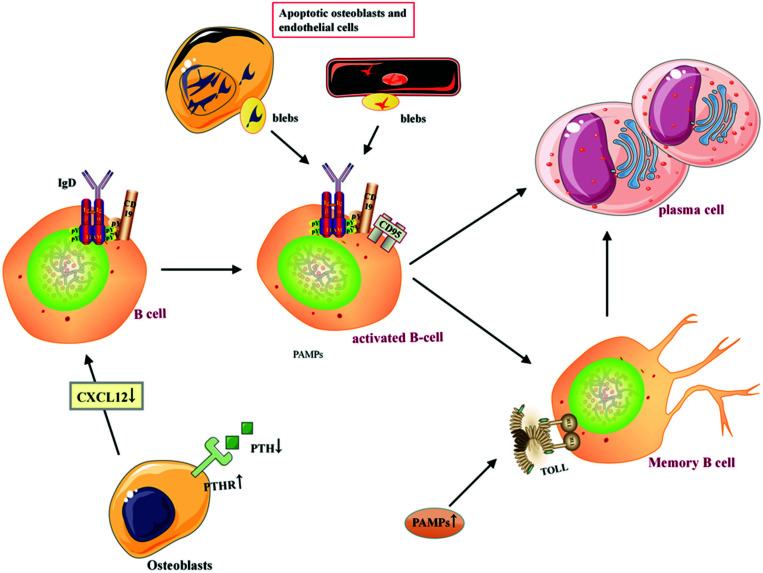
Diagrammatic representation of the mechanism of action of B cells in SANFH.

**Fig. (17):**
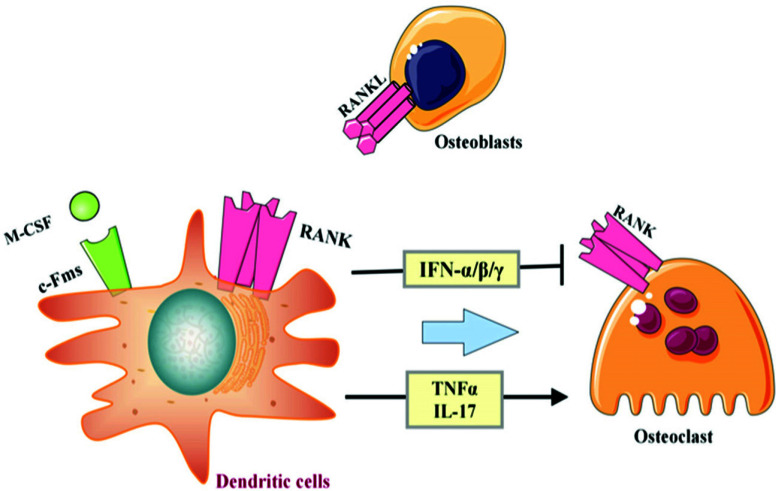
The mechanism of action of dendritic cells in SANFH.

**Table 1:** Basic information on key genes.

**mRNA**	**Related Description**	**Degree Value**	**Difference Multiple**	**Trend**
EP300	E1A binding protein P300	16	0.58	Up
TRAF6	TNF receptor associated factor 6	12	0.51	Up
STAT1	Signal transducer and activator of transcription 1	11	0.52	Up
JAK1	Janus kinase 1	9	0.55	Up
CASP8	Caspase 8	9	0.57	Up
JAK2	Janus kinase 2	8	0.63	Up

## Data Availability

The data, used in this study can be obtained from the corresponding author upon request.

## LIST OF ABBREVIATIONS

ANFH or ONFH: Avascular Necrosis of the Femoral Head
BMSCs: Bone Marrow Mesenchymal Stem Cells
CASP8: Caspase 8
CSF: Granulocyte Colony-stimulating Factor
CXCL12 and SDF-1: C-X-C Motif Chemokine Ligand 12
DAMPs: Damage-associated Molecular Patterns
EP300: E1A Binding Protein P300
GC: Glucocorticoid
GO: Gene Ontology
HE: Hematoxylin and Eosin
IFN-β: Interferon Beta
IRAK1: Interleukin-1 Receptor-associated Kinase-1
JAK1: Janus Kinase 1
JAK2: Janus Kinase 2
KEGG: Kyoto Encyclopedia of Genes and Genomes
MMP-13: Matrix Metalloproteinase-13
MyD88: Myeloid Differentiation Factor 88
NETs: Neutrophil Traps
NLRP3: NOD-like Receptor Pyrin Domain-containing 3
PAMPs: Pathogen-associated Molecular Patterns
PPI: Protein-protein Interaction
PRRs: Pattern Recognition Receptors
RANKL: Receptor Activator of Nuclear Factor Kappa-B Ligand
SANFH: Steroid-associated Necrosis of the Femoral Head
STAT1: Signal Transducer and Activator of transcription 1
TAK1: Transforming Growth Factor-beta-activated Kinase 1
TFH: Follicular Helper T Cells
TGF-β: Transforming Growth Factor Beta
TGFR: TGF-β Receptors
Th: Helper T Cells
TRAF6: TNF Receptor-associated Factor 6
Tregs: Regulatory T Cells
